# Metatarsal Shaft Fracture with Associated Metatarsophalangeal Joint Dislocation

**DOI:** 10.1155/2016/9629585

**Published:** 2016-08-11

**Authors:** Taranjit Singh Tung

**Affiliations:** Division of Orthopaedic Surgery, Boundary Trails Health Centre, P.O. Box 2000, Station Main, Winkler, MB, Canada R6W 1H8

## Abstract

Metatarsophalangeal joint dislocations of lesser toes are often seen in the setting of severe claw toes. Traumatic irreducible dislocations have been reported in rare cases following both low-energy and high-energy injuries to the forefoot. In this case report, I present a previously unreported association of a metatarsal shaft fracture with metatarsophalangeal joint dislocation of a lesser toe.

## 1. Introduction

Irreducible traumatic metatarsophalangeal (MTP) joint dislocations have been reported in various case reports following both low-energy and high-energy injuries. Often these dislocations are dorsal in direction and associated with plantar plate injuries [1–4].

I report a case of traumatic lesser metatarsal (MT) fracture and MTP joint dislocation in an adult male after a motor vehicle accident. This case illustrates a previously unreported association between MT shaft fracture and MTP joint stability and failure of early recognition of radiographic subluxation at the MTP joint and offers a systematic approach to a symptomatic patient with a semiacute MTP dislocation with MT malunion.

## 2. Case Report

**Figure 1:**
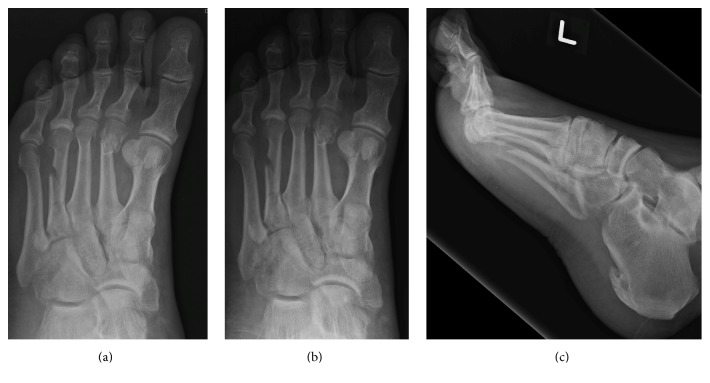
Dorsoplantar (a), oblique (b), and lateral (c) radiographs of the left foot at initial presentation.

This is the case of a 56-year-old man who was involved in a motor vehicle accident resulting in a right ulna fracture and multiple fractures in his left foot. The fractures in his left foot included a second-metatarsal (MT) neck fracture, proximal third-MT fracture, and a comminuted fracture of the fourth MT (Figure 1). Subluxation of the fourth metatarsophalangeal (MTP) joint was not recognized by the treating physician; hence between the patient and his treating physician nonoperative treatment for his injuries was decided upon.

His past medical history was significant for West Nile encephalopathy resulting in residual right upper extremity weakness and spasms.

**Figure 2:**
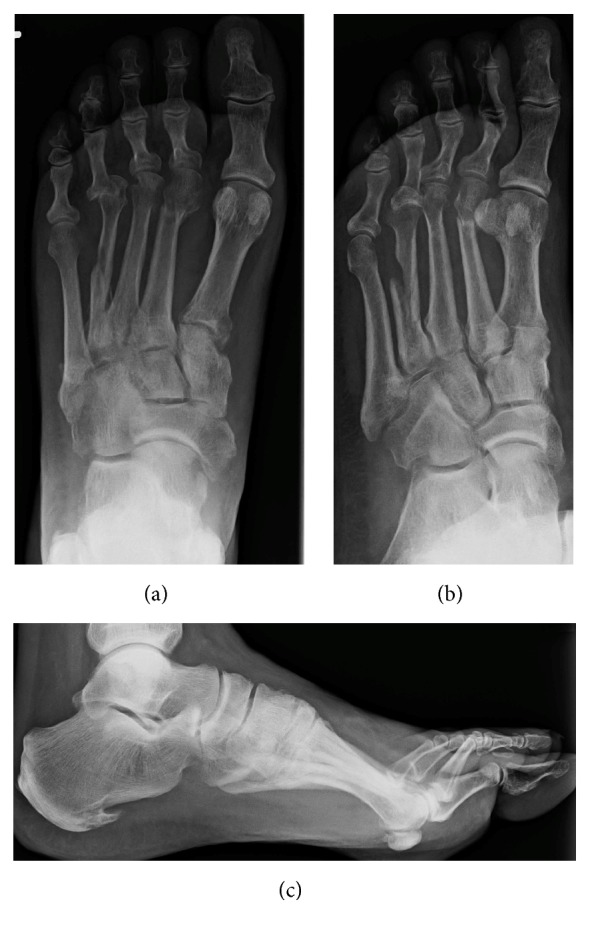
Preoperative dorsoplantar (a), oblique (b), and lateral (c) radiographs of the left foot showing malunion at fracture site, resulting in a lengthened fourth MT, and a dorsal dislocation at the fourth MTP joint.

**Figure 3:**
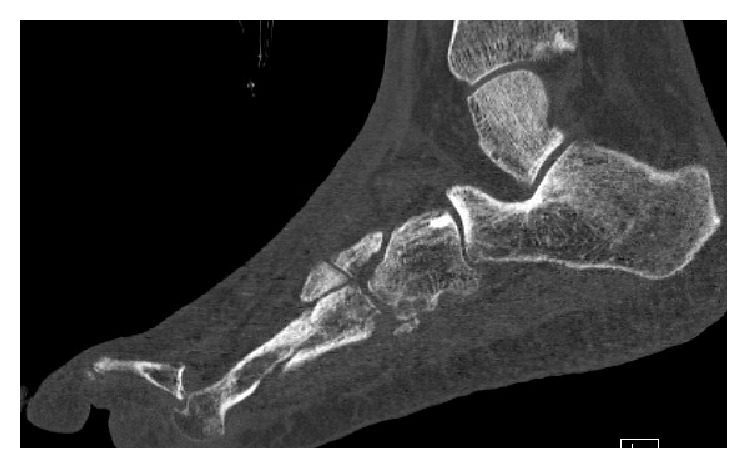
CT scan at the level of the left 4th toe illustrating fracture malunion and dorsal dislocation at the MTP joint.

The patient was referred to my fracture clinic nine weeks after his injury with ongoing pain in the left foot at the known fracture sites but also at the level of his fourth MTP joint. Radiographs and CT scan imaging of his foot revealed healing fractures of the 2nd and 3rd MTs in acceptable position, a partial malunion of the 4th MT, and a dorsal dislocation of the 4th MTP joint (Figures 2 and 3). On careful evaluation of his images it was evident that he had disrupted the normal lateral descending cascade of the lesser metatarsals [5]—with his 4th metatarsal having been lengthened via the malunion site to almost the same length as his 3rd metatarsal. An in-depth discussion regarding treatment options was held with the patient, and he opted for surgical intervention. An informed consent for the procedure and publication of this case report was obtained from the patient.

**Figure 4:**
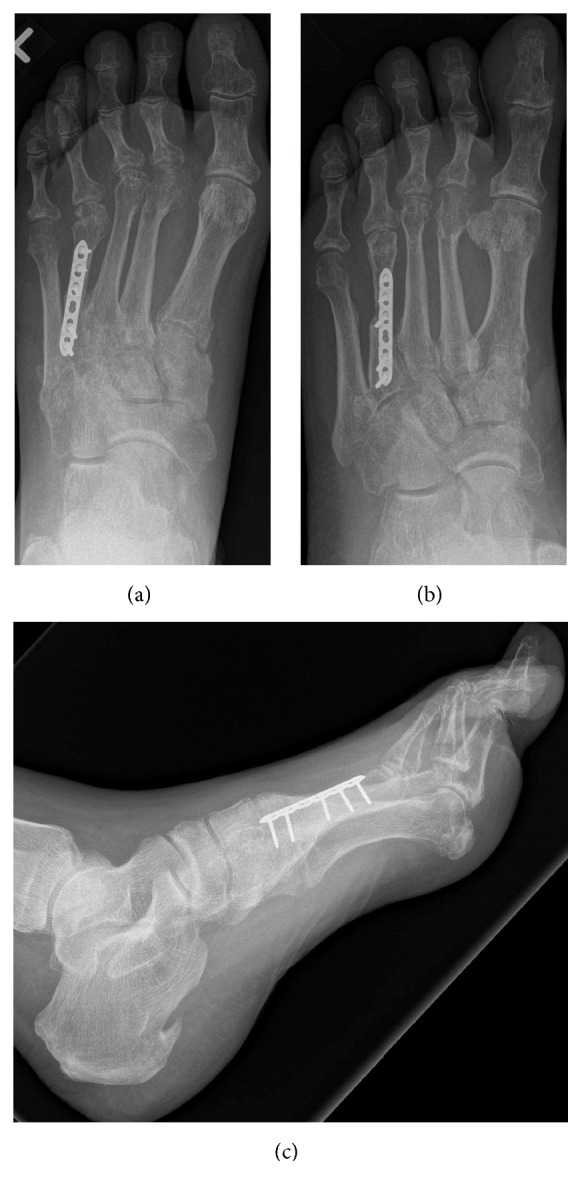
Final dorsoplantar (a), oblique (b), and lateral (c) radiographs showing fusion at fracture site, shortening of fourth MT, and maintained reduction at the fourth MTP joint.

Patient received a general anesthetic. He was positioned supinely on the operating table and a thigh tourniquet was used. Attempt at closed reduction of the 4th MTP joint was unsuccessful. Dorsal longitudinal incision centered over the 4th MT was utilized to get to the fracture site. Three main fragments were noted. Some healing in extension was noted on the dorsal surface between a couple of the fragments, but a free floating plantar fragment was also seen. Even after the fragments were mobilized, closed reduction of the 4th MTP joint was not possible. The incision was thus extended distally, the extensor tendons were mobilized, a dorsal capsulotomy was performed, and the collateral ligaments were released off the MT head. Even with that, a reduction of the dislocated joint was not possible. So at this point I decided to shorten the MT through the fracture site and secure it with a minifragment locking plate (Synthes, Mississauga, Ontario). A lag screw through the plate was utilized to secure the plantar fragment, which was sculptured to fit in. The 4th MTP joint was then reduced. The plantar plate was inspected and noted to be intact. The position of the reduced MTP joint was maintained with a 1.6 mm Kirschner wire. Layered closure of the incision was performed prior to immobilizing the foot in a cast boot. Three doses of antibiotics were administered postoperatively, and the patient was discharged home the following day. Stitches were taken out about three weeks later, and the pin was removed about 6 weeks postoperatively. Heel weight-bearing was allowed at 6 weeks and full-weight-bearing was allowed at 12 weeks. Patient did have delayed wound healing but made excellent recovery, and, at final follow-up eight months after surgery, his 4th MT was fully healed, 4th MTP joint maintained a reduced position, there was some evidence of disuse osteopenia but no evidence of avascular necrosis (AVN) of the MT head, and he was back to his preinjury level of activity (Figure 4).

## 3. Discussion

MTP dislocations are often noted in 2nd toes in association with hallux valgus deformities, especially in patients with a long second MT relative to the 1st MT [6, 7]. In rheumatoid feet multiple claw toe deformities and associated MTP dislocations are common [8]. Irreducible traumatic MTP dislocations have been reported in various case reports. These are usually dorsal in direction and often a result of axial load applied to hyperextended toes [1–4]. Plantar dislocations are rare and usually due to dorsally directed force on the plantar aspect of metatarsal heads [9]. This paper presents a previously undescribed injury comprising both MT shaft fracture and MTP joint subluxation and ultimately dislocation of a lesser toe. Due to the initial displacement, the fracture partially healed, nonanatomically, with lengthening through the fracture site—effectively increasing the length of the 4th MT and thus disrupting the normal metatarsal parabola. Failure of early recognition and management of the subtle subluxation gradually caused the subluxed 4th proximal phalanx to ultimately dislocate dorsally on the MT head. Attempt at closed reduction of the MTP joint at nine weeks proved to be futile without first addressing the MT length. Weil's osteotomy is commonly used to shorten the MT length to aid in correction of severe claw toe deformities, allowing reduction of the MTP joint [10]. Similar shortening osteotomies can also be performed through the shaft of the MT [11]. Using this latter principle in this case of irreducible MTP joint following traumatic lengthening of the MT, shortening through the fracture site offered an attractive option to address the MT length and thereby facilitate reduction at the MTP joint. Unlike most of the other reported cases of traumatic MTP dislocation, a tear of the plantar plate was not noted, likely due to force dissipation through not just the MTP joint but also the MT shaft. It is possible that the MT fractured first, and as it was displaced it resulted in lengthening of the MT, which, in turn, disrupted the normal metatarsal parabola, causing the MTP joint to initially sublux and then be ultimately dislocated dorsally. Thus correction of MT length was integral in achieving reduction of this lesser toe MTP dislocation. Immobilization of the reduced joint with a K-wire to allow for soft tissue scarring proved to be beneficial. To avoid pin breakage and minimize motion at the fracture/osteotomy site the patient was kept non-weight-bearing in a cast boot for 6 weeks, prior to progressing his weight-bearing.

## 4. Conclusion

This case illustrates an unusual injury, MT shaft fracture with associated MTP subluxation—ultimately resulting into MTP joint dislocation, the need for early recognition of such an injury, and a practical, systematic approach to management of a semiacute MTP dislocation following MT malunion—with shortening of the MT, dorsal capsulotomy, collateral ligament release, reduction of MTP joint, maintenance of reduction with K-wire fixation, and immobilization till soft tissues scar in, to achieve an excellent clinical outcome.
